# Beauty isn’t everything: An agent-based model of imperfect food acceptance and market utility balance

**DOI:** 10.1371/journal.pone.0334504

**Published:** 2025-12-08

**Authors:** Yara Khaluf, Ilona E. de Hooge

**Affiliations:** 1 Computational Intelligence Group, Department of Computer Science, Vrije Universiteit Amsterdam, Amsterdam, Netherlands; 2 Marketing and Consumer Behaviour Group, Department of Social Sciences, Wageningen University, Wageningen, Netherlands; Lusofona University of Humanities and Technologies: Universidade Lusofona de Humanidades e Tecnologias, PORTUGAL

## Abstract

Imperfect or suboptimal foods—cosmetically flawed yet nutritionally sound—are frequently discarded across the food supply chain, contributing significantly to global food waste. Although farmers, retailers, and consumers all influence this waste dynamic, existing research often treats their behaviors in isolation and fails to capture the evolving, systemic interplay among them. This study presents a novel agent-based model simulating interactions between farmers, retailers, and consumers in local produce markets to examine how preferences, stocking strategies, and marketing interventions shape market behavior over time. The model integrates behavioral mechanisms such as the mere-exposure effect and Prospect Theory to realistically represent consumer adaptation and decision-making. Through simulations across varying market configurations, we identify critical points in consumer behavior and market-wide utility. Our findings reveal that promoting imperfect foods can lead to substantial utility gains for both consumers and retailers—particularly when retailers stock high levels of imperfect products consistently over time. These results emerge from simulations showing that the mere-exposure effect drives consumer preference shifts even in the absence of marketing, enabling widespread acceptance under certain stocking strategies.

## Introduction

Of all the sustainability challenges currently threatening the planet, the waste of perfectly edible foods stands out as one of the most irrational. While the global food production system requires significant natural resources and accounts for approximately one-third of all greenhouse gas emissions, about one-third to one-half of all food produced is ultimately wasted [1]. Reducing food waste has therefore been identified as a critical global action for building a more sustainable future [2]. An essential proportion of this food waste across all stages of the supply chain concerns so-called imperfect or suboptimal foods [3]. Imperfect or suboptimal foods are foods that meet all quality and safety standards but deviate from product specifications on the basis of their appearance (e.g., shape, size) [4]. Once foods deviate from these cosmetic specifications, they are often removed from the production line and discarded [5,6].

At the heart of imperfect food waste is a systemic misalignment among supply chain stakeholders. Farmers often express willingness to sell imperfect produce but face constraints due to perceived reluctance from retailers and consumers. Retailers, in turn, report a willingness to stock these items but cite inconsistent supply and low consumer demand as limiting factors [5,7]. Consumers frequently reject imperfect products based on aesthetic norms, unfamiliarity, and perceived lower quality, with rejection rates varying by demographics and product attributes such as appearance and price [8,9]. Limited retail availability further suppresses exposure and acceptance.

Despite this interdependence, most existing research has examined these actors in isolation and treated their behaviors as static. While some studies include multiple stakeholders, they do not capture how preferences and decisions evolve dynamically over time [5,7]. A system-wide, temporal approach is needed to reveal how actor decisions influence one another, how consumer exposure shapes preferences, and how interventions like awareness-raising or naturalness-focused marketing strategies may generate unintended effects or feedback loops across the supply chain.

To understand and potentially influence these dynamics, this study introduces an agent-based model that simulates the interactions among farmers, retailers, and consumers within local produce markets. The model captures the economic behaviors of supply-side agents and the psychological and behavioral complexity of demand-side actors. It particularly emphasizes the mere-exposure effect—the psychological phenomenon whereby repeated exposure to a stimulus increases an individual’s preference for it—as a key driver in shaping consumer attitudes toward imperfect produce [10]. In addition, consumer decision-making is modeled using principles from Prospect Theory, capturing asymmetric responses to gains and losses that better reflect real-world choice behaviors under uncertainty [11]. Prospect Theory posits that individuals evaluate outcomes relative to a reference point rather than in absolute terms. Gains produce diminishing satisfaction (concave utility), while losses are experienced more intensely—a phenomenon known as loss aversion.

Our model investigates how variations in consumer preferences, awareness levels, and retailer stocking strategies affect market outcomes such as utility, satisfaction, waste, and retailer-switching behavior. Scenarios are constructed to reflect diverse market contexts, including populations with widely distributed, skewed-to-imperfect, and skewed-to-perfect preferences. The model also incorporates consumer persistence in searching for preferred products, retailer nature-friendliness, and targeted marketing strategies (awareness vs. naturalness), allowing for rich behavioral diversity and adaptive feedback across the supply chain. Through this analysis, we identify critical tipping points in consumer behavior and explore the long-term benefits of stocking imperfect products—not only as a sustainability initiative but also as a viable economic strategy for retailers [9]. Ultimately, the model offers a dynamic lens for evaluating how market-wide utility balances shift under various interventions, revealing trade-offs between economic outcomes and waste reduction that static analyses overlook.

Our work offers new insights into how strategic decisions at the retailer level can reshape consumer preferences over time. The findings have implications for designing policies and marketing interventions aimed at fostering environmentally conscious and economically efficient food systems.

## Materials and methods

We propose an agent-based model [12] to study the market dynamics in a supply chain of farmers, retailers, and consumers in vegetable/fruit local markets. Hence, our model incorporates three types of agents—farmers, retailers, and consumers—each interacting in a system governed by economic incentives, behavioral dynamics, and environmental considerations.

### Farmers

are tasked with producing vegetables/fruits that are divided into two categories: perfect and imperfect. Their production reflects the natural variability of agricultural outputs, with imperfect products constituting a randomly determined proportion of the total production. Farmers sell their produce to retailers, aiming to maximize their income while minimizing unsold stock. Their utility function *U*_*F*_ is formalized as:


$$U_{F}=\text{Income}=Q_{\text{perfect}}\cdot P_{\text{perfect}}+Q_{\text{imperfect}}\cdot P_{\text{imperfect}},$$

where *Q* represents quantities sold and *P* the corresponding prices.

### Retailers

Act as intermediaries between farmers and consumers, purchasing products from the former and selling them to the latter. Their primary aim is to maximize utility, which in this context represents profit derived from sales revenue minus the cost of unsold goods. To achieve this in our model, retailers implement marketing strategies—either awareness, a strategy focused on making people aware of the sustainability issues surrounding imperfect products, or naturalness, a strategy highlighting the naturalness or authenticity of the imperfect products—in an attempt to influence consumer purchasing decisions. Additionally, each retailer is assigned a “nature-friendliness" attribute, representing their internal commitment to sustainability. This attribute guides their decisions on the proportion of imperfect products to stock. Retailer utility *U*_*R*_ is modeled as:


$$U_{R}=\text{Revenue}-W_{\text{cost}},$$

where revenue derives from product sales, and $W_{\text{cost}}$ accounts for unsold stock valued at its purchase price from farmers:


$$W_{\text{cost}}=({\text{Stock}}_{\text{perfect}}\cdot {\text{Purchase Price}}_{\text{perfect}})+({\text{Stock}}_{\text{imperfect}}\cdot {\text{Purchase Price}}_{\text{imperfect}})$$

### Consumers

In the model, consumers are characterized by a set of traits that govern their behavior and decision-making processes. As consumers don’t always have a single, clear-cut preference, each consumer is assigned a **preference score** for both perfect ($p_{\text{perfect}}$) and imperfect ($p_{\text{imperfect}}$) products, reflecting their intrinsic attitudes toward these categories. Beyond preferences, consumers exhibit **persistence**, a trait representing their determination to pursue preferred products even when unavailable. Higher persistence values indicate a greater likelihood of switching retailers or seeking alternatives to align purchases with preferences. Additionally, consumers respond variably to retailer marketing strategies. Their **response to awareness** and **response to naturalness** capture their sensitivity to marketing efforts. These response levels introduce situational flexibility into the model, allowing consumers to adapt their decisions in the presence of targeted strategies. Together, these traits create a detailed representation of consumer behavior, balancing intrinsic preferences, situational influences, and individual persistence.

Consumers’ preference scores may change over time as a result of the **mere-exposure effect** [13]. This posits that repeated exposure to a stimulus increases an individual’s preference for it. In the model, consumers’ preferences for perfect or imperfect products evolve based on their availability in retailer stock, at every retailer visit. The preference adjustment is expressed as:


$$p_{\text{imperfect}}=\min(p_{\text{imperfect}}+\sigma ,1),\text{where }\sigma =\;0.05\text{ in our simulations}$$

These updates enable the model to capture the gradual shift in consumer attitudes driven by familiarity, even in the absence of direct persuasion.

### Consumer purchase decision rule

Each consumer computes an inclination score for both perfect and imperfect products at a given time step, influenced by intrinsic preferences and the retailer’s marketing strategy:


$$I_{\text{perfect}}=p_{\text{perfect}},\;I_{\text{imperfect}}=w_{\text{pref}}\cdot p_{\text{imperfect}}+(1-w_{\text{pref}})\cdot s,$$

where $p_{\text{perfect}}$ and $p_{\text{imperfect}}$ represent the consumer’s current preferences, $w_{\text{pref}}\in [0,1]$ is the consumer’s weight for preference versus strategy influence, and *s* is the strategy influence defined as:


$$s=\left\{ \begin{array}{cc} \text{response\_to\_awareness}, & \text{if retailer uses awareness strategy},\\ \text{response\_to\_naturalness}, & \text{if retailer uses naturalness strategy},\\ 0, & \text{otherwise}. \end{array}\right.$$

The purchase decision is made as follows:


$$\text{Decision}=\left\{\begin{array}{lc} “\text{imperfect}”, & \text{if}\;{\text{I}}_{\text{imperfect}}\;>\;{\text{I}}_{\text{perfect}}\;\text{and stoc}{\text{k}}_{\text{imperfect}}\;>\;0,\\ “\text{perfect}”, & \text{if}\;{\text{I}}_{\text{perfect}}\;\geq \;{\text{I}}_{\text{imperfect}}\;\text{and stoc}{\text{k}}_{\text{perfect}}\;>\;0,\\ “\text{switch}”, & \text{if preferred product is unavailable and random}()\;<\;\text{persistence},\\ “\text{fallback}”, & \text{if preferred product is unavailable and random}()\;\geq \;\text{persistence},\\ \text{None}, & \text{if no product is available or budget is insufficient} \end{array}\right)$$

After the inclination towards both types of products is computed, they are compared and if the consumer’s inclination towards imperfect products is higher and they are available by the retailer, the consumer opts for the imperfect option. Conversely, if the consumer’s inclination towards perfect products is higher or if imperfect products are unavailable, the consumer selects perfect products if they are in stock. In cases where neither the preferred product nor a compelling alternative is available, **fallback behaviors** emerge based on the consumer’s persistence trait and intrinsic preference. If the consumer has a higher preference for perfect products but these are out of stock, their persistence determines the subsequent action. For consumers with high persistence, those switch to another retailer in search of the preferred product, reflecting a determined pursuit of their preference. Conversely, for consumers with low persistence, those accept the less-preferred alternative at the current retailer—purchasing imperfect products—if these are available in stock. For consumers with a preference for imperfect products they purchase perfect products from the current retailer when imperfect products are out of stock. The **retailer-switching** mechanism enables consumers to keep moving to the next unvisited retailer as long as the preferred type of product is not found, until all retailers are visited. Then consumers buy from the last-visited retailer.

After purchasing a product, the utility of the consumer is defined by two key factors: the spent budget and satisfaction with the purchased product. A higher proportion of the budget spent decreases utility, reflecting the consumer’s reduced economic flexibility, while satisfaction with the product, determined by how well it aligns with their preferences, increases utility. Consumer satisfaction is evaluated using **Prospect Theory** [14], which accounts for the asymmetric valuation of gains and losses relative to a reference point. Satisfaction, *S*, is modeled based on whether the purchased product aligns with the consumer’s preference:


$$S=\left\{ \begin{array}{cc} p_{\text{product}}^{a} & \text{if preference alignment is a gain,}\\ -\lambda \cdot |p_{\text{product}}{|}^{b} & \text{if preference alignment is a loss.} \end{array}\right.$$

Here, *a* and *b* are parameters capturing diminishing sensitivity to gains and losses, while *λ* represents loss aversion, reflecting the greater psychological weight of losses compared to equivalent gains.

In this model, market-wide utility balance is understood as a simulation-derived condition in which the average utilities of consumers, retailers, and farmers converge and stabilize over time. Rather than representing a formal mathematical equilibrium, it reflects a dynamic steady state where utility trajectories for each agent class level off.

### Analytical metrics

We use the following metrics to analyze the micro and macro dynamics of the market system.

### 1. Preference distribution percentage

The proportion of consumers who prefer imperfect/perfect products compared to those who prefer the other product category at a given time step. This metric illustrates the population-level distribution of preferences for perfect and imperfect products, allowing analysis of shifts in consumer behavior over time for both categories.


$$\text{Imperfect\_Preference\_Percentage}=\left(\frac{\text{Number of consumers where }p_{\text{imperfect}}>p_{\text{perfect}}}{\text{Total number of consumers}}\right)\cdot 100$$

Perfect_Preference_Percentage=100−Imperfect_Preference_Percentage

For each time step, the model compares the preference scores $p_{\text{imperfect}}$ and $p_{\text{perfect}}$ for all consumers. It calculates the percentage of consumers preferring imperfect products ($p_{\text{imperfect}}>p_{\text{perfect}}$) and assigns the remaining percentage to those preferring perfect products ($p_{\text{perfect}}>p_{\text{imperfect}}$).

### 2. Average preference

This metric quantifies the average preference for perfect and imperfect products across all consumers at a given time step. It highlights the mean population-level tendency toward either category, reflecting cumulative exposure effects.


$$\text{Average\_Preference\_Imperfect}=\frac{\sum_{i=1}^{N}p_{\text{imperfect},i}}{N}$$

$$\text{Average\_Preference\_Perfect}=\frac{\sum_{i=1}^{N}p_{\text{perfect},i}}{N}$$

Here, $p_{\text{imperfect},i}$ and $p_{\text{perfect},i}$ represent the individual preference scores of consumer *i* for imperfect and perfect products, respectively, and *N* is the total number of consumers.

### 3. Total waste quantity

This metric reflects the total quantity of unsold perfect and imperfect products accumulated across all retailers at a given time step. It helps assess inefficiencies in inventory management.


$$\text{Waste\_Perfect}=\sum_{j=1}^{M}{\text{Stock}}_{\text{perfect},j}$$

$$\text{Waste\_Imperfect}=\sum_{j=1}^{M}{\text{Stock}}_{\text{imperfect},j}$$

Here, ${\text{Stock}}_{\text{perfect},j}$ and ${\text{Stock}}_{\text{imperfect},j}$ represent the remaining stock of perfect and imperfect products for retailer *j*, and *M* is the total number of retailers.

### 4. Average retailer switches

This metric tracks the average number of retailer switches consumers make over time, averaged across all simulation runs. It provides insights into the persistence and adaptability of consumers in finding their preferred products under varying market conditions.


$$\text{Average\_Retailer\_Switches}=\frac{\sum_{r=1}^{R}{\text{Switches}}_{r,t}}{R}$$

Here, ${\text{Switches}}_{r,t}$ represents the total number of retailer switches made by consumers at time step *t* during simulation run *r*, and *R* is the total number of simulation runs. This calculation provides a step-by-step view of consumer switching behavior averaged over different realizations of the market dynamics.

### 5. Satisfaction and variance in satisfaction

Satisfaction measures the alignment between purchased products and consumer preferences, while variance captures the disparity in satisfaction across consumers.

Satisfaction *S* is computed using Prospect Theory:


$$\text{Satisfaction}=\left\{ \begin{array}{cc} p_{\text{product}}^{a} & \text{if preference alignment is a gain,}\\ -\lambda \cdot |p_{\text{product}}{|}^{b} & \text{if preference alignment is a loss.} \end{array}\right.$$

Here, $p_{\text{product}}$ is the preference for the purchased product, *a* and *b* represent diminishing sensitivity parameters, and λ accounts for loss aversion.

Variance in satisfaction is computed as:


$$\text{Variance\_Satisfaction}=\frac{\sum_{i=1}^{N}(S_{i}-\mu_{S}{)}^{2}}{N}$$

where *S*_*i*_ is the satisfaction of consumer *i*, and $\mu_{S}$ is the mean satisfaction across all consumers.

## Results

The results of the study are presented across several simulation configurations designed to capture a range of market dynamics and behavioral scenarios. These configurations vary key parameters, including consumer intrinsic preferences, awareness levels, and retailer stocking strategies for imperfect products (IP). A detailed summary of the three scenarios and their parameter settings is provided in Table 1. Specifically, the study explores (i) a population with distributed preferences across the full spectrum of product quality, (ii) a population skewed toward imperfect products, and (iii) a population skewed toward perfect products. In each scenario, retailer stocking strategies vary between low and high levels of imperfect product availability, enabling an examination of how preference alignment, exposure effects, and satisfaction evolve under different market conditions.

**Table 1:** Summary of simulation configurations and key parameter ranges. IP = Imperfect Products.

Scenario	Preference Range	Awareness Range	Retailer IP Stocking	Persistence
Distributed	[0.1,1.0] uniform	[0.0,1.0] uniform	L: [0.1,0.2] / H: [0.7,0.9]	[0.1,1.0] uniform
Skewed to Imperfect	Imperfect: [0.8,1.0]; Perfect: [0.1,0.2]	[0.0,1.0] uniform	L: [0.1,0.2] / H: [0.7,0.9]	[0.1,1.0] uniform
Skewed to Perfect	Perfect: [0.8,1.0]; Imperfect: [0.1,0.2]	[0.0,1.0] uniform	L: [0.1,0.2] / H: [0.7,0.9]	[0.1,1.0] uniform

### Populations with widely-distributed preferences

**Fig 1:**
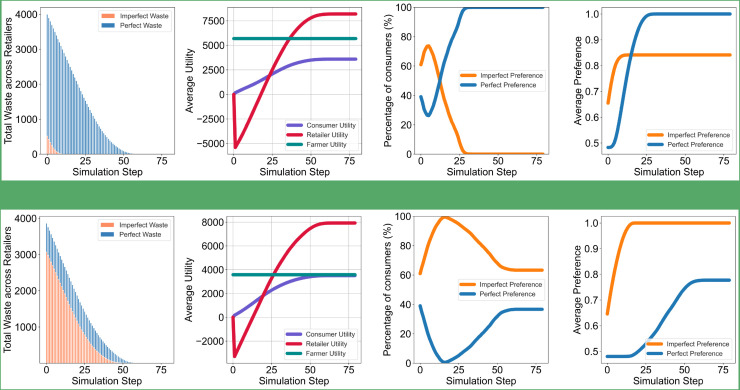
Average consumer preferences and utility outcomes over time in a population with widely distributed intrinsic preferences for perfect and imperfect products. The simulation compares two retailer stocking strategies: low (top row) vs. high (bottom row) percentages of imperfect products (IP). In the low-IP condition, consumer preference shifts toward perfect products due to higher availability, driven by the mere-exposure effect. In contrast, high-IP stocking reinforces imperfect product preference and maintains higher consumer satisfaction and alignment with stock. Farmer utility is highest in the low-IP condition due to consistent demand for perfect products.

The results depicted in Fig 1 highlight dynamics in consumer and market behaviors when perfect and imperfect intrinsic preferences are distributed between [0.1–1]. When retailers maintain a low intake of imperfect products (IP) in their stock (upper line), the consumers’ instinctive preference for imperfect products, initially slightly higher than their preference for perfect products, declines rapidly. This shift is driven by the mere-exposure effect, which increases the preference for perfect products due to their higher availability. Consequently, a crossover occurs between the average preferences for perfect and imperfect products at approximately step 18, where the percentage of consumers preferring each category also switches.

In contrast, when retailers stock a high percentage of imperfect products (lower line), the average imperfect preference of consumers continues to grow from its initial state, again driven by the mere-exposure effect. This growth stabilizes at its maximum around step 18, after which the average perfect preference begins to increase. Although the percentage of consumers preferring perfect products rises beyond this point, the percentage favoring imperfect products remains higher than those favoring perfect products until the end of the simulation.

Despite the differences in retailer stocking strategies, the utility levels for retailers and consumers converge, showing no significant divergence between the low and high imperfect stock strategies. However, farmer utility exhibits a marked difference: it is higher when retailers prioritize perfect products in their stock. This is because farmers produce a larger quantity of perfect products, allowing them to meet retailer demands effectively. Conversely, lower imperfect stock percentages lead retailers to source from multiple farmers, reducing the individual utility of any single farmer. This lower farmer utility does not result from pricing mechanisms or product rejection, as product prices are fixed and all products are assumed to meet quality standards. Instead, it stems from a structural characteristic of the simulation: when retailers increase their stocking of imperfect products, they tend to diversify purchases across multiple farmers to fulfill those needs. This fragmentation of demand reduces the total volume sold per farmer, thereby lowering their income and utility.

Finally, the waste dynamics differ remarkably across stocking strategies. In the low-IP condition, perfect product waste accumulates more rapidly over time as consumer preferences shift and stock mismatches emerge. Conversely, high-IP stocking results in higher initial waste of imperfect products, but this stabilizes as consumer preferences adapt, ultimately reducing overall waste compared to the low-IP scenario.

**Fig 2:**
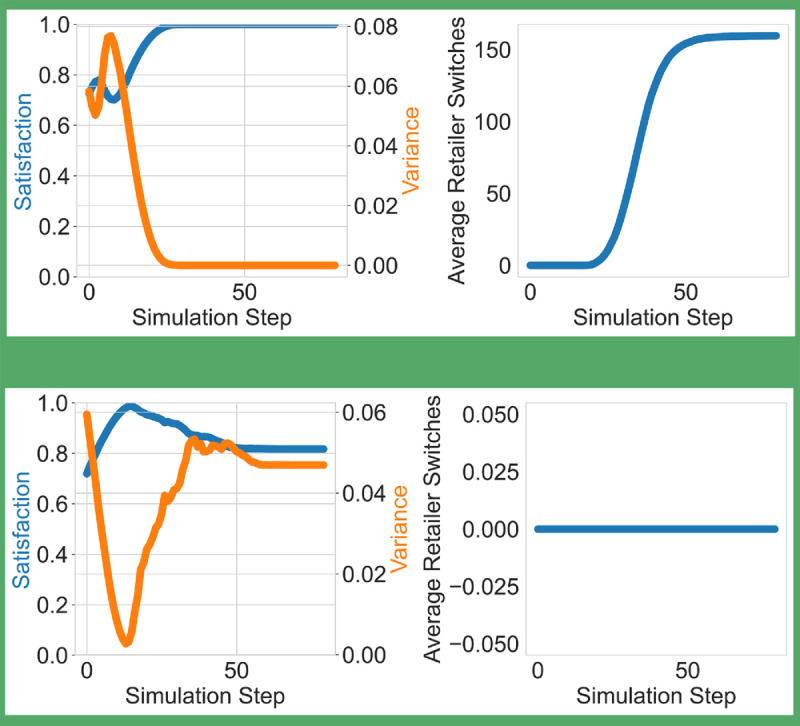
Dynamics of consumer satisfaction and retailer-switching behavior in the widely distributed preference scenario. In the low-IP condition (top row), a rising mismatch between consumer preferences and available stock leads to increased retailer switching and a temporary drop in satisfaction. In contrast, the high-IP (bottom row) condition sustains high satisfaction and near-zero switching due to better alignment between stock and preference. Satisfaction variability increases in both cases as preferences diversify over time.

At the consumer level (Fig 2), a key behavioral dynamic emerges after step 20: as the average consumer preference for perfect products surpasses that for imperfect ones—driven by the mere-exposure effect and increased visibility of perfect products—consumers begin prioritizing perfect products. However, by this time, the retailer’s stock of perfect products has significantly declined, falling to nearly half of its initial level in the low-imperfect-stock scenario (Fig 1, upper line). This mismatch between rising preference and limited availability triggers a spike in retailer-switching behavior, particularly among consumers with high persistence (distributed between 0.1 and 1), who are more likely to search across retailers. As a result, the average number of retailer switches increases markedly.

In contrast, under the high-imperfect-stock scenario (Fig 1, lower line), the average preference for imperfect products remains dominant throughout the simulation. This alignment between consumer preferences and retailer stock ensures that most consumers find their desired products at the first retailer visited, keeping retailer switching negligible—effectively zero. The behavioral stability highlights the efficiency of aligning product availability with consumer demand.

Regarding satisfaction, when retailers stock a low percentage of imperfect products (Fig 1, upper line), initial satisfaction is below 1 due to the mismatch between higher imperfect preferences and the overwhelming availability of perfect products. Once the average preference for perfect products surpasses that for imperfect products (around step 18), satisfaction rises rapidly to its maximum.

Conversely, in the high-imperfect-stock case, satisfaction increases early in the simulation alongside the rise in average imperfect preferences (Fig 2, lower line). However, as the proportion of consumers favoring perfect products grows while imperfect products remain predominant in stock (Fig 1, lower line), satisfaction declines to approximately 0.8. When consumer preferences become more evenly split between the two categories, satisfaction variance increases, reflecting heterogeneous consumer experiences.

### Populations with skewed preferences

**Fig 3:**
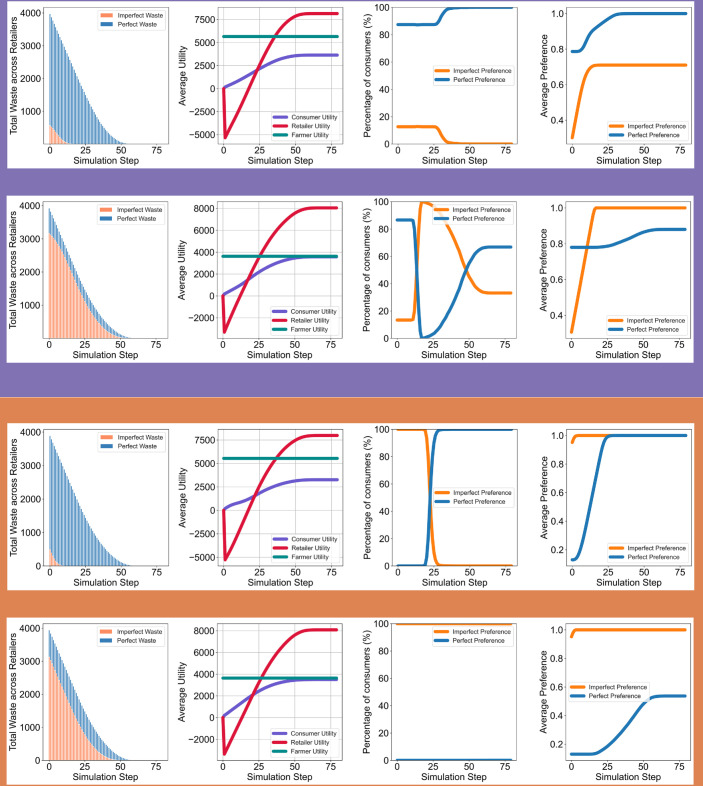
Evolution of average consumer preferences and product dominance in populations with skewed intrinsic preferences. In 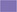 we show results for a population skewed toward perfect products, while in 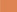 we represent a population skewed toward imperfect products. Each scenario compares low vs. high percentages of imperfect product (IP) stocking by retailers. In perfect-preferring populations, low-IP stocking (top row) reinforces existing preferences and minimizes volatility, whereas high-IP stocking (bottom row) initially shifts preference toward imperfect products, before reverting. In imperfect-preferring populations, preference for imperfect products remains dominant under high-IP stocking (bottom row). However, under low-IP stocking (top row), imperfect items quickly run out, and continued exposure to perfect products causes a shift in preferences—eventually leading to a crossover where perfect product preferences surpass imperfect ones, despite the population’s initial skew.

Fig 3 illustrates the dynamics of consumer behavior and market outcomes in populations with a skewed preference for perfect products, under two contrasting retailer stocking strategies: low (upper line) and high (lower line) percentages of imperfect products.

When retailers maintain a small percentage of imperfect products in their stock (Fig 3 - upper line), the initial average preference for perfect products starts higher than that for imperfect products. Over time, this preference for perfect products grows further due to the mere-exposure effect, reaching its maximum by approximately step 20. This increase occurs as the limited stock of imperfect products is consumed, leading to the absence of further exposure to imperfect products and a continued dominance of perfect products in retailer stock. Consequently, the percentage of consumers with perfect preferences reaches its peak, while those with imperfect preferences diminish.

In contrast, when retailers stock a high percentage of imperfect products, the initial average preference for perfect products is quickly surpassed by the preference for imperfect products due to increased exposure to the latter. This shift results in a switch in consumer dominance, with the percentage of consumers preferring imperfect products overtaking those preferring perfect products around step 20. While the average preference for imperfect products stabilizes at its maximum at this point, the average preference for perfect products continues to grow until approximately step 50. This leads to another shift in dominance, where the percentage of consumers preferring perfect products eventually exceeds those favoring imperfect products.

Such a double-switch pattern in consumer dominance does not occur in populations with a skewed preference for imperfect products. In those cases, when retailers stock a low percentage of imperfect products, the average preference for perfect products grows rapidly due to the high availability of perfect products, causing a single switch in dominance at around step 22. However, when retailers stock a high percentage of imperfect products, the average preference and percentage of consumers favoring imperfect products remain dominant throughout the simulation.

The trends in utility and waste closely mirror those observed in populations with widely distributed preferences. Retailer and consumer utilities reach similar levels under both low and high imperfect product stocking strategies. Farmer utility, however, is higher when retailers stock a large percentage of perfect products. This is because farmers produce more perfect products, ensuring consistent supply to meet retailer demands and reducing the need for retailers to switch suppliers.

Waste patterns in these skewed-preference populations also reveal important contrasts between stocking strategies. When consumers predominantly prefer perfect products, high-IP stocking leads to persistent waste of imperfect items that are not purchased despite repeated exposure. When consumers predominantly prefer imperfect products, high-IP stocking results in higher initial waste of imperfect items, which gradually stabilizes as demand aligns with availability. Conversely, low-IP stocking generates less early waste but leads to increasing waste of perfect products over time as consumer preferences remain skewed toward imperfect produce and stock mismatches accumulate.

**Fig 4:**
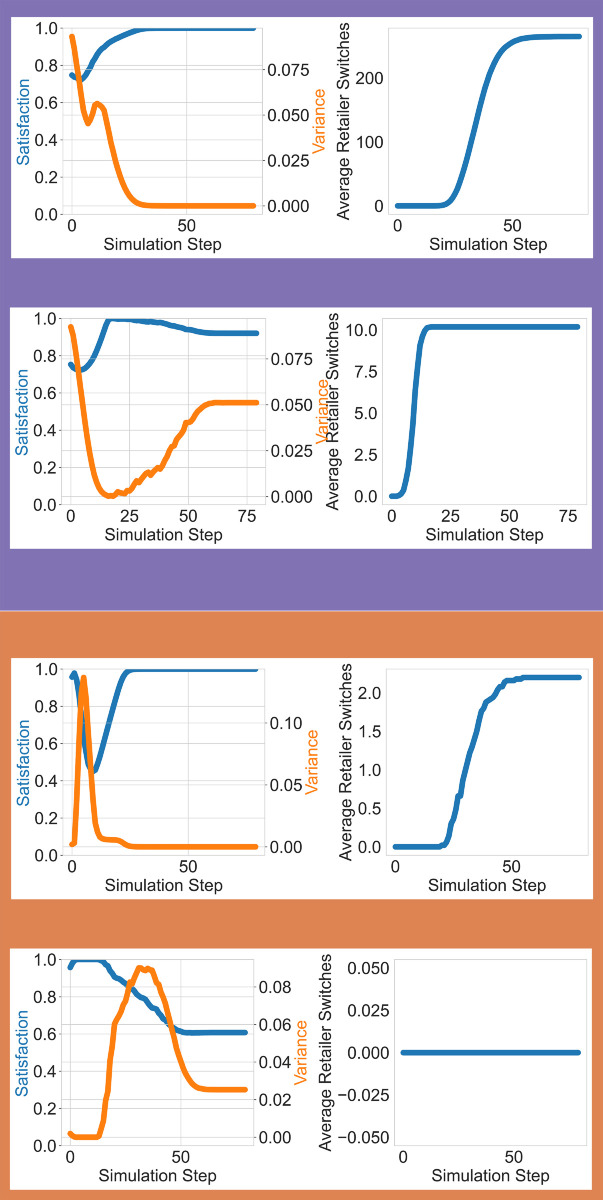
Retailer-switching behavior and consumer satisfaction across populations with skewed intrinsic preferences. In 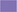 we show results for populations skewed toward perfect products, whereas in 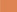 we show results for populations skewed toward imperfect products. Under low-IP stocking (top row), perfect products become increasingly scarce, prompting consumers—especially those with high persistence—to switch retailers more frequently in search of their preferred items. In contrast, high-IP stocking (bottom row) in this group misaligns with preferences but maintains product availability, resulting in fewer switches but lower satisfaction and increased variance. In imperfect-preferring populations 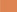, retailer-switching behavior is minimal regardless of the stocking strategy, as consumers with imperfect preferences don’t switch retailers when imperfect products are not available. Satisfaction is highest when retailer stock aligns with consumer preferences; misalignment leads to lower satisfaction and increased variance. In both skewed populations, preference shifts over time—especially under exposure effects—can create divergence between stock availability and evolving demand.

Retailer-switching behavior varies significantly depending on the population’s preference skew and the retailer’s stocking strategy Fig 4. For populations skewed toward perfect preferences, when retailers maintain low imperfect stocking, the average preference for perfect products remains higher throughout the simulation. However, as perfect product availability decreases over time, some consumers fail to find their preferred products, prompting them to visit additional retailers. This leads to an increase in average retailer visits, particularly for consumers with high persistence (distributed between [0.1, 1]). On the other hand, when retailers maintain high imperfect stocking, the average preference for imperfect products surpasses perfect products within the first 10 steps and remains dominant for the remainder of the simulation. This dominance ensures that consumers with imperfect preferences do not need to switch retailers, stabilizing retailer visits at lower levels (around 8).

For populations skewed toward imperfect preferences, retailer-switching behavior is minimal regardless of the stocking strategy. The average preference for imperfect products remains dominant, ensuring a higher percentage of consumers with imperfect preference who doesn’t switch retailer.

Consumer satisfaction vary across populations and stocking strategies. In populations skewed to perfect preferences, when retailers maintain high perfect stocking, satisfaction grows to its maximum value as perfect product availability aligns with consumer preferences. Variance between consumers decreases to its minimum, reflecting a uniform experience. When retailers stock a high percentage of imperfect products, satisfaction remains high initially, but variance increases as the average preference for perfect products begins to grow. The mismatch between increasing perfect preferences and the dominance of imperfect products drives this variability.

In populations skewed toward imperfect preferences, when retailers stock a high percentage of perfect products, satisfaction drops initially due to the dominance of perfect products, which are less preferred by the population. Variance increases during this period of misalignment but stabilizes once the average preference for perfect products reaches its maximum. Conversely, when retailers stock a high percentage of imperfect products, satisfaction decreases as the average preference for perfect products grows. Variance remains high during this period, reflecting the divergence between the availability of imperfect products and the increasing average preference for perfect products.

## Discussion and conclusion

This study examined the dynamic interactions between farmers, retailers, and consumers in the context of imperfect food products, using an agent-based modeling approach to simulate market-wide behavior over time. Although previous research has documented barriers to the adoption and acceptance of imperfect foods across different supply chain actors, it has primarily done so through static analyses or actor-specific studies. In contrast, the present work adopts a dynamic systems perspective to assess the feasibility and sustainability of integrating imperfect products into the regular food supply.

Our findings contribute to the existing literature by demonstrating that imperfect foods can be successfully incorporated into the market ecosystem—but only under specific conditions. In particular, the model shows that extensive and sustained stocking of imperfect products by retailers can gradually lead to increased consumer acceptance via the mere-exposure effect. Over time, this can bring retailers and consumers into a state of market-wide utility balance, in which both parties achieve high levels of satisfaction and utility. However, a key boundary condition emerged: farmers consistently attain lower utility in scenarios where imperfect products are introduced. This reduction in utility is not due to margin compression or product rejection—both of which are absent from the model—but rather from reduced quantities sold per farmer. When demand for imperfect products increases, retailers source from a broader set of farmers, distributing their purchases more widely. This diversification, while beneficial for supply availability and risk distribution, dilutes individual farmer sales and contributes to their consistently lower utility levels in the simulation.

A noteworthy insight from the model is that the cumulative effect of mere exposure may rival or even exceed the influence of explicit marketing strategies. This has been revealed using a set of control simulations with no marketing strategies. Those have yielded preference shifts and utility patterns comparable to those in awareness and naturalness conditions, indicating that mere exposure alone plays a dominant role in influencing consumer behavior. This suggests that consistent stocking practices could, in themselves, be powerful tools for market transformation—an idea with substantial practical relevance for retailers and policymakers. Nonetheless, it remains an open question whether these effects would hold under more complex representations of consumer memory, such as category-specific recall of marketed claims.

Theoretically, this research is among the first to investigate the long-term, system-wide consequences of introducing imperfect foods into mainstream retail. This methodology enabled the modeling of multiple actor types, each with distinct behavioral rules and interdependencies. It also allowed us to observe emergent feedback loops, such as how increased stocking of imperfect products led to greater consumer exposure, which in turn reinforced acceptance and influenced future purchasing decisions. Adaptations were observed in both consumer preferences—via the mere-exposure mechanism—and in retailer strategies, as some retailers began shifting their imperfect/perfect stock balance in response to changes in demand and sales performance. The concept of Market-Wide Utility Balance introduced in this work provides a new lens through which to evaluate the collective outcomes of sustainability-oriented interventions. It emphasizes that a solution perceived as beneficial from one stakeholder’s perspective may yield suboptimal or even negative effects for others—especially when measured over time.

Several limitations of the current model merit discussion. First, while the model incorporates key psychological mechanisms (e.g., the mere-exposure effect and Prospect Theory), it does not reflect the full heterogeneity of real-world consumers. Future extensions could integrate consumer segmentation to account for differences in motivation, awareness, and price sensitivity, as identified in recent food marketing literature.

Second, our operationalization of utility for farmers and retailers focused primarily on sales-based revenue outcomes. In practice, additional factors—such as logistical complexity, transportation costs, reputational risk, and brand positioning—may influence decision-making, particularly when stocking imperfect products. For instance, retailers targeting premium segments may avoid imperfect products not due to lack of demand, but because of concerns about brand coherence. Incorporating these dimensions into future models would provide a more comprehensive picture of the utility trade-offs involved. It is also important to note that the current model does not account for retailer segmentation or branding strategies. In real-world markets, retailers targeting premium segments may intentionally avoid stocking imperfect products—not due to lack of supply or profitability, but to preserve brand coherence and consumer perceptions of quality. While our model includes a nature-friendliness parameter to simulate sustainability-oriented stocking behavior, it assumes that all retailers operate under similar economic logic. This represents a simplification. Incorporating differentiated retailer types with distinct strategic objectives—such as premium versus discount positioning—would allow for more realistic modeling of heterogeneous market behaviors and might reveal new system-level trade-offs. We identify this as a valuable direction for future work.

Finally, while the model simulated structural marketing interventions (e.g., awareness and authenticity strategies), it did not model the effects of consumer memory or advertising exposure over time. Extending the consumer architecture to account for differentiated responses to messaging, or for social and normative influences, could significantly enrich the predictive power of the model.

While the model was developed in the context of consumer retail, its mechanisms—such as preference shifts through repeated exposure—can extend to institutional settings like school meal programs, food banks, or government procurement schemes. In these environments, consistent stocking of imperfect foods could normalize their acceptance and reduce rejection-driven waste. Our model thus offers a framework for designing structural interventions beyond traditional markets.

The present findings highlight that structural exposure mechanisms—such as consistent stocking of imperfect products—may offer a more reliable path to consumer acceptance than messaging strategies alone. This insight has actionable implications for retailers, who can leverage the mere-exposure effect without relying exclusively on persuasion-based marketing. However, the benefits of such market transformations are not uniformly distributed. Farmers, in particular, may be disadvantaged by fragmented sourcing patterns that arise under high-imperfect-stock scenarios.

Future research should also investigate the role of differentiated retailer strategies, perishability dynamics, and consumer segmentation in shaping long-term market stability and stakeholder equity. By advancing models that account for both behavioral and structural complexity, we can better inform the design of interventions that promote sustainable and inclusive food systems.
